# Latent class analysis of attention and white matter correlation in children with attention-deficit/hyperactivity disorder

**DOI:** 10.1590/1414-431X20187653

**Published:** 2018-10-04

**Authors:** A.S.U. Rossi, L.M. Moura, M.C. Miranda, M. Muszkat, C.B. Mello, O.F.A. Bueno

**Affiliations:** 1 Departamento de Psicobiologia, Universidade Federal de São Paulo, São Paulo, SP, Brasil; 2 Centro de Matemática, Computação e Cognição, Universidade Federal do ABC, SP, Brasil; 3 Departamento de Psicologia, Curso de Pós-graduação em Psicossomática, Universidade Ibirapuera, São Paulo, SP, Brasil; 4 Programa de Educação e Saúde da Infância e Adolescência, Universidade Federal de São Paulo, Guarulhos, SP, Brasil

**Keywords:** Attention-deficit/hyperactivity disorder, Attention, White matter, Pattern recognition, Diffusion tensor imaging

## Abstract

This study aimed to explore attentional patterns among children with inattentive attention-deficit/hyperactivity disorder (ADHD-I) and children with typical development (TD), using a latent class analysis (LCA). Patterns of brain connectivity were also explored. The sample comprised 29 ADHD-I and 29 TD matched children. An LCA was conducted to reclassify subjects according to their attentional performance, considering cognitive measures of attention and behavioral symptoms, regardless of group of origin. The new clusters were then compared in respect to brain white matter measurements (extracted from diffusion tensor imaging). Participants were rearranged in 2 new latent classes, according to their performance in an attention task and the results of behavioral scales, resulting in groups with more homogeneous attentional profiles. A comparison of the 2 new classes using the white matter measurements revealed increased fractional anisotropy in the left inferior fronto-occipital fasciculus and left inferior longitudinal fasciculus for the class composed by participants with a higher risk of attentional problems. The findings indicated that it was possible to observe variability regarding neuropsychological profile, accompanied by underpinning neurobiological differences, even among individuals with the same disorder subtype - inattentive ADHD. This specific data-driven clustering analysis may help to enhance understanding of the pathophysiology of the disorder's phenotypes.

## Introduction

Studies exploring cognitive, behavioral, or emotional patterns, as well as neurobiological or genetic traits among diagnostic conditions and typically developing individuals are becoming a trend in neuropsychiatry (1–4). In 2009, the National Institute of Mental Health (NIMH) launched the Research Domain Criteria (RDoC) project, to develop, for research purposes, new ways of classifying mental disorders based on dimensions of observable behavior and neurobiological measures. This research framework is a step towards a dimensional and integrative view of psychopathologies, linking behavior and cognitive variables to underlying neurobiological or genetic systems, cutting across diagnostic categories and improving understanding of the complex relationships between these factors (5).

Growing evidence has established that attention-deficit/hyperactivity disorder (ADHD) is a highly heterogeneous condition, with a diverse presentation and multiple variables pointing to its determination, being classified as a dimensional diagnosis (6).

In order to investigate this heterogeneity, van Hulst et al. (4) investigated neuropsychological subtypes in individuals with ADHD and typically developing (TD) controls. Three subgroups with differing cognitive profiles were identified, based on their performance in a battery of tests related to cognitive control, timing, and reward systems, supporting a multiple pathway model of ADHD.

Fair et al. (1) also evaluated a large dataset of ADHD and TD individuals in respect to working memory, temporal information processing, response speed, variability and inhibition, interference control, arousal, and activation. Several cognitive tests were adopted, among them a version of the Continuous Performance test (CPT). Four different neuropsychological profiles were found among ADHD participants and, analogously, among controls.

Similarly, the CPT, more specifically Conners’ Continuous Performance test (CCPT-II) (7), was used in a pattern recognition study conducted with healthy controls, individuals with ADHD, bipolar disorder (BD) and ADHD+BD. Based on CCPT performance, the sample was clustered into two new groups, regardless of the original diagnosis, demonstrating that CCPT-II variables may be accurate and useful measures to classify subjects according to their cognitive performance (2).

Indeed, the CPT is a common and reliable neuropsychological instrument for the evaluation of sustained attention and other attention subcomponents (8). Attention is a complex cognitive ability and encompasses multiple interconnected brain regions (9), with many different white matter fibers connecting these regions.

Diffusion tensor imaging (DTI) seems to be a very promising method of investigating white matter microstructural abnormalities that could underpin mechanisms of inattention as it allows a shift in perspective in the investigation of the pathophysiology of ADHD, with less focus on brain regions and more emphasis on brain network organization (10).

The sensitivity of DTI – and of its measurements, such as fractional anisotropy (FA) – in identifying microstructural tissue characteristics makes this technique a powerful and sensitive instrument to investigate changes in white matter microstructure in clinical conditions such as ADHD (10,11). FA represents the amount of hindrance/restriction experienced by water molecules along the direction of white fibers (11) and has been the DTI measurement most frequently adopted in studies of ADHD subjects (12). FA values are modulated by many factors of functional relevance, including, but not limited to fiber diameter, fiber density, myelination, and membrane permeability (11).

Previous studies have found that individuals with ADHD present either higher or lower FA values, indicating fiber abnormalities that might negatively impact their cognitive functioning (10,13). As pointed out by Thomason and Thompson (10), ADHD is a rare example where higher FA is indicative of pathology, possibly representing either less branching (i.e., more net coherence in a singular direction) or compensatory mechanisms. However, the high heterogeneity observed in the disorder has resulted in inconsistent findings among different studies. Indeed, a previous analysis conducted with the same sample used in the present study did not reveal any differences in FA measurements when ADHD and TD individuals were compared (14).

We believed that if these same subjects were analyzed through a dimensional analysis, considering their cognitive profile rather than their original diagnosis, the results in respect to underlying white matter measurements could be different. Thus, we aimed to investigate attentional patterns among the ADHD-I and TD children in order to identify more homogeneous groups in regard to attentional performance through an LCA. Moreover, based on the identified groups, we intended to investigate possible differences regarding underlying brain characteristics, as recommended by Fair et al. (1).

To the best of our knowledge, very few studies have compared and analyzed the relationship between behavioral or emotional measures and physiological or neuroimaging data through a dimensional analysis in respect to ADHD (3,15). The present study is the first to integrate both cognitive and behavioral measures with neuroimaging data through an LCA.

We hypothesized that individuals would be rearranged regarding attentional functioning patterns and that the new groups would be associated to differences in the FA measure of their brain tracts. Attentional performance, measured by some indexes of the CCPT-II, digit span test, and by behavioral measures, was established as an independent variable, while FA values were considered dependent variables.

## Material and Methods

### Participants

Recruited participants included 29 ADHD-I (ADHD of inattentive type) children aged 7–15 years and 29 TD children matched by age, sex, and type of high school attended (public or private), recruited using a purposive sampling method. ADHD-I patients were referred from public and private centers, where they had been diagnosed by an expert ADHD clinician based on the best estimate diagnostic procedure. A clinical examination, personal interview, family history from family informants, and the DSM-IV criteria were considered, as well as the results from a neuropsychological assessment, which was also part of the diagnosis protocol of these centers. ADHD participants, at the time of diagnosis, presented six or more inattention symptoms, and up to five hyperactivity/impulsivity symptoms, characterizing the predominantly inattentive type. All of the TD children were recruited from the community.

Eligibility criteria were an estimated intelligence quotient (IQ) of at least 85 on the Wechsler Intelligence Scale for Children, third edition (WISC-III) (16), measured by Block Design and Vocabulary subtests, and no previous history of neurological diseases. Seven children from the TD group and nine from the ADHD-I group were excluded from imaging analysis due to excessive motion during imaging or due to artifacts. Thus, 22 TD and 20 ADHD-I images met the quality criteria for processing, and comprised the sample used in the neuroimaging stage of analysis.

Three of the ADHD-I children had been taking methylphenidate for a few months, but stopped the medication at least 24 hours before testing. This research was approved by the Ethics Committee of the Universidade Federal de São Paulo and was carried out in accordance with the ethical standards as laid down in the 1964 Declaration of Helsinki and its later amendments or comparable ethical standards. The parents provided written informed consent for all of the participants. The children provided verbal or written informed assent.

### Instruments

Participants were submitted to a broad neuropsychological evaluation, which included an examination of the subject's intellectual level, a computerized attention test, a working memory test, and other tests to assess other cognitive functions not analyzed in this study, such as visual constructive functions, visual memory, verbal fluency, decision making, and academic performance. This evaluation was used to better delineate their cognitive profile and to identify other diagnostic conditions, such as dyslexia.

As measures of interest, variables extracted from a computerized attention test – CCPT-II (7) – and from a working memory performance test – digit span (16) – were selected. Primary caretakers completed a sociodemographic questionnaire, the Child Behavioral Checklist (CBCL) (17) and the SNAP-IV Teacher and Parent Rating scale (18). The measures of interest used in the LCA were chosen based on accumulated evidence in respect to measures that have been consistently found to be impaired in subjects with ADHD. Thus, as CPT's and working memory measures have assumed a prominent role as potential endophenotype indicators of the disorder and are correlated to biological markers, these measures were selected as variables to compose the LCA models. Moreover, as behavioral measures of attentional functioning, CBCL scales were also included since they have been shown to be a highly discriminative instrument in ADHD evaluation. Thus, the following measures were adopted to conduct the LCA.

Five CCPT-II measures were selected following a non-structured review of CCPT studies with ADHD participants, as they were found to be very sensitive in identifying nuances in attentional profiles (7). The indexes were: Omissions, Variability, Hit Reaction Time Interstimulus Interval (Hit RT ISI), Hit Reaction Time (Hit RT), and Hit Reaction Time Block Change (Hit RT BC). Raw values provided by the test for each of these indexes were considered in order to compare participants' performances.

Backward digit span test from the WISC-III (16) was applied as another measure of attentional control (19). Two trials were administered for each span (sequence length ranging from 2 to 8), in reverse order. Two errors in the same span prompted interruption of the task and, thus, the higher span achieved was considered. The span achieved was standardized into z-score measures, adopting Brazilian norms, as described in data analysis.

Attention Problems subscale and the Emotional Self-Regulation Index (20) of the CBCL were both included as indicative of attentional performance and self-regulation in daily situations (17). Caretakers' responses to items related to these scales were scored by summing 0-1-2 ratings. Raw scores were transformed into T-scores by the CBCL software, based on normative data. T-scores less than 65 were considered in the normal range, T-scores ranging from 65–70 were considered to be borderline clinical, and T-scores above 70 were in the clinical range (17).

### DTI image acquisition protocol

A 2-D MR-DTI sequence (TR=6500 ms, TE=95 ms, flip angle=90 degrees, matrix size=128 × 128, NEX=1, FOV=230 mm, 12 directions, b=1000 m/s^2^, thickness=4 mm with space gap between slices =0.8 mm, yielding 30 slices) was acquired using a 3.0 -T, 43 mT/m gradient MR system (Magnetom Verio, Siemens Medical Systems, Germany) for all subjects. All images had their quality verified, rated based on an initial visual inspection, and followed by the inspection and detection of artifacts such as geometrical distortions and susceptibility effects, such as motion.

### DTI images post-processing

NifTi images were processed using the FSL platform (21) using the following the steps: correction of eddy currents, skull extraction using BET tool, and a FDT tool to generate FA maps. All the FA maps were then merged into a 4-D image using tract-based spatial statistics (TBSS) (21). Since the sample was composed of children, the most appropriate choice for registration was to align each FA image to every other, identifying the most representative FA map, to use it as a reference image of the group. A mean FA skeleton was generated and the FA values of the most relevant tracts from the spatially normalized FA map of each subject were then projected onto this skeleton using a threshold of 0.3. Permutation-based non-parametric inference was applied to unsmoothed statistical maps using 10,000 permutations and the cluster-like structures were enhanced using the threshold-free cluster enhancement (TFCE) algorithm at P level <0.05 (family-wise error (FWE) correction).

A 4-D FA skeletonized image was used, with all mean skeletons of each subject projected and the skeleton mask of the group, to run an automated region of interest (ROI) extraction, using R project 3.0.3. The extraction of the tracts was based on the 20 tracts of the Johns Hopkins University DTI white-matter tractography atlas (22), resulting in a mean FA value for each tract of each subject.

### Data analysis

Demographic characteristics and the intellectual level of the sample were statistically analyzed using a Student's *t*-test and chi-squared test (statistical software SPSS version 19.0; IBM, USA). Raw neuropsychological measures were standardized into z-score measures, adopting Brazilian norms (23,24). The CBCL Attention Problem subscale was incorporated utilizing a t-score measure, generated by CBCL rating software (17).

LCA was used to identify a latent organizing principle (i.e., classes) for a complex set of empirically observed continuous data. Categorical latent variables are not directly observed and are defined as those in which qualitative differences exist between groups of people or objects (25). Three different models were built to reorganize the participants, considering six different continuously observed performance indicators, extracted from the CCPT-II, backward digit span task, and the CBCL.

Three LCA models were established to estimate how many classes underlaid the six indicators selected. Clinical issues and the following statistical indexes were used to guide the decision on the best number of classes: Akaike information criterion (AIC), Bayesian information criterion (BIC), sample size-adjusted BIC (ssaBIC), Vuong-Lo-Mendell-Rubin (VLMR) test (using TECH 11 in Mplus) and the parametric bootstrapped likelihood ratio test (using TECH 14 in Mplus). The last two tests compared the fit of the model with the currently chosen number of classes (K) to the fit of a model with K-1 classes. Additionally, the quality of individual items (variable-specific entropy contribution) was calculated (26). Convergence validity of the best model/class-solution was calculated, testing some covariates (ADHD clinical diagnosis by a trained specialist, type of school [public *vs* private], gender, and age), as possible predictors of latent classes via the three-step approach, developed by Vermunt (27), and entered in Mplus.

The best solution found was used to predict the 20 FA mean values, via Lanza's method (28), which was adopted as an approach to calculate differences in FA means between the classes. Since there was a P value associated with each FA (resulting in 20 P values), and being an exploratory procedure, the Benjamini-Hochberg procedure for false discovery rate was applied in respect to the 20 P values (29). As not all 58 subjects had neuroimaging FA outcomes, Lanza's method was employed with 42 subjects, using Bayes theorem, where the joint distribution of the latent class variable and the distal variable were represented as a regression of the latent class variable, conditional on the distal variable, combined with the marginal distribution of the distal variable (the 20 DTI parameters). The smaller size of the sample at this analysis reduced the statistical power of the estimation of likely difference in FA means, but it did not impact the previously achieved best class solution because Lanza's method does not allow for the distal outcome to dramatically change the class membership for individual observations. The idea behind the method is that after the LCA model is estimated, an auxiliary model is estimated where the 20 distal outcomes are used as a latent class predictor within a multinomial logistic regression in addition to the original measurement LCA model. As implemented in Mplus, Lanza's method used approximate standard errors for continuous distal outcomes by estimating the mean and variance within each group as well as the within class sample size. Standard errors were then computed as if the mean estimate was the sample mean. For both continuous and categorical distal outcomes, Mplus computed an overall test of association using Wald's test, as well as pairwise class comparisons between the auxiliary variable means and probabilities. These analyses adopted a significance level of 0.05.

## Results

The TD and ADHD-I groups did not differ significantly regarding age, gender, or type of school attended (Table 1). The TD participants had significantly higher intellectual functioning (*t*(55)=–2,47; P=0.02), but mean IQs were above average score of IQ scale for both groups (i.e., above 110).

**Table 1:** Sociodemographic characteristics of the sample groups.

Characteristics	TD group (n = 29)	ADHD-I group (n = 29)	Test statistics	P
Age (years, mean ± SD)	10.1±1.6	10.1±1.9	T = -0.73	0.94
Gender (% male)	65.5%	69.0%	χ^2^ = 0.78	0.78
Type of school (% public)	62.1%	50.0%	χ^2^ = 0.84	0.36
Estimated IQ (mean±SD)	120.2±14.9	110.6±14.9	T = -3.26	0.02

TD: typical development; ADHD-I: attention-deficit/hyperactivity disorder of inattentive type; IQ: intelligence quotient; T: *t*-test; χ^2^: chi-squared test.

### Latent class analysis - the best model and best class solution

The whole sample (n=58) was submitted to LCA, regardless of the origin group, in order to identify subsets of individuals with more similar attentional patterns. Three different models were built, each one comprising six continuously observed performance variables. Model 1 comprised three CCPT-II indexes (omission, Hit RT, and Hit RT ISI), backward digit span, the attention problems subscale and emotional self-regulation index from the CBCL, balancing cognitive measures extracted from different tasks, and a behavioral variable. Model 2 consisted of four CCPT-II indexes (Omission, Hit RT, Hit RT ISI, and Hit RT Block Change) and the two CBCL behavioral measures, associating only CCPT-II variables with behavioral indicators. Finally, Model 3 was composed exclusively of direct attentional variables, namely the five cognitive ones extracted using CCPT-II (Omission, Hit RT, Hit RT ISI, Hit RT Block Change, and Variability) and a behavioral measure related to attention performance in daily situations (the CBCL attention problems subscale). The fit indices for the three models and the class solutions are shown in Table 2. Regarding statistical criteria, the lower the AIC, BIC, and ssaBIC values, the better the model. Not only the fit indices, but also the clinical reasoning is equally important in the selection of the best model to regroup participants.

**Table 2:** Model fit indexes.

Model	Number of classes	AIC	BIC	ssaBIC	Entropy	Class 1	Class 2	Class 3	Class 4	VLMRLR	PBLRT
M1	2 classes	1650.0	1689.1	1629.4	0.92	33	25			0.02	<0.001
	3 classes	1604.3	1657.9	1576.1	0.95	22	29	7		0.21	<0.001
	4 classes	1579.9	1647.9	1544.1	0.96	28	17	5	8	0.33	<0.001
M2	2 classes	1615.8	1654.9	1595.2	0.92	35	23			0.18	<0.001
	3 classes	1576.5	1630.1	1548.4	0.95	8	15	35		0.14	<0.001
	4 classes	*	*	*	*						
M3	2 classes	1363.4	1402.5	13.8	0.93	19	39			0.11	<0.001
	3 classes	1304.4	1357.9	1276.2	0.95	15	38	5		0.06	<0.001
	4 classes	*	*	*	*						

AIC: Akaike information criterion; BIC: Bayesian information criterion; ssaBIC: Sample size-adjusted BIC; VLMRLR: Vuong-Lo-Mendell-Rubin likelihood ratio; PBLRT: parametric bootstrapped likelihood ratio test. *Model did not converge.

Model 3, with a three- and a two-class solution, presented the lowest AIC, BIC, and ssaBIC. The three-class solution was not a suitable distribution for the current study due to the small number of participants in one of the classes (n=5), which would not allow the white matter analysis to be carried out appropriately. Therefore, the Model 3/two-class solution was selected as the most appropriate to reorganize the subjects according to their attentional profiles.

**Figure 1:**
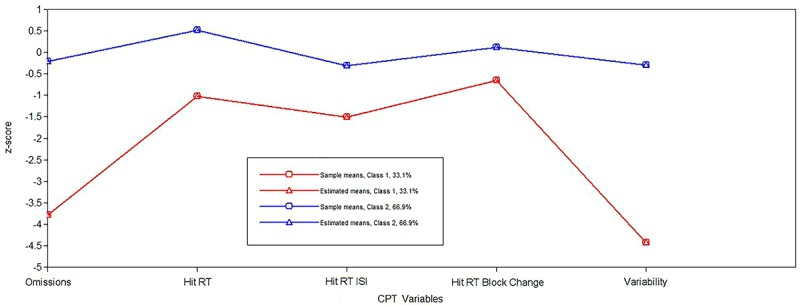
Z-score means for the five Conners’ Continuous Performance test (CCPT-II) indexes within each latent class for Model 3/Two-class solution. Child Behavioral Checklist attention problems subscale revealed T-score values of 56.82 for the normal development class (blue line) and of 72.19 for the ultra-high risk class (red line). Hit RT: Hit Reaction Time; Hit RT ISI: Hit Reaction Time Interstimulus Interval.

This model/class solution comprised two groups, which were called: the ultra-high risk for attentional problems group (HR-class) (33.1% of the sample; ADHD-I n=19; TD n=0), shown in Figure 1 as Class 1 (red line), and the normal development of attentional functions group (ND-class) (66.9% of the sample, ADHD-I n=11; TD n=28), shown in Figure 1 as Class 2 (blue line). The groups did not differ in respect to age (*t*(56)=0.89, P=0.14), gender (χ^2^(1)=0.02, P=0.89), or estimated IQ (*t*(55)=-1.68, P=0.10), avoiding the possibility of attentional differences being due to IQ differences (see more details in Supplementary Table S1). About 36.6% of the ADHD-I individuals showed probability to be classified as belonging to the ND-class.

Figure 1 shows how participants were grouped according to their performance in the five CCPT-II variables adopted. The ND-class presented an average performance in all the cognitive measures adopted (Z-scores varying between −0.35 and +0.5), while the HR-class was characterized by presenting a deficient performance in all the cognitive indexes. Z-scores obtained for each class varied as follows: Omissions (ND=–0.21; HR=–3.78); Hit RT (ND=0.51; HR=–1.02); Hit RT ISI (ND=–0.31; HR=–1.51); Hit RT BC (ND=0.11; HR=–0.66), and Variability (ND=–0.30; HR=–4.43).

Similarly, responses to the CBCL attention problem subscale revealed a score suggestive of an absence of attentional problems for the ND-class (T-score=56.82, SD=2.15), while they were suggestive of attentional problems at a clinical level for the HR-class (T-score=72.19, SD=1.21).

Moreover, in the latent classes, in regard to the severity of the symptoms of inattention and hyperactivity/impulsivity of the subjects of each class (that is, the members who were likely to be in each of the classes), it was noted that 77% of those in the HR-class presented 6 or more symptoms of inattention at considerable levels (quite a bit or very much), according to responses given on the SNAP scale. Approximately 32% of those in the ND-class also presented 6 or more symptoms of inattention at the same levels. Thus, although there was a predominance of symptoms of inattention among the HR-class, these symptoms were also observed at significant levels among the ND-class.

In respect to comorbidities, before the LCA analysis, four ADHD participants presented dyslexia and one anxiety disorder. No comorbidities were found among the TD subjects. After the LCA division, two of the participants with dyslexia and the one with anxiety were placed in the HR-class. The remaining dyslexic patients were placed in the ND-class. Only one of these latter participants had his neuroimaging excluded due to poor quality.

### Entropy contribution

All six continuous indicators of Model 3 (the five CCPT-II indexes and the CBCL attention problem subscale) were compared regarding their capacity to discriminate the new subgroups. The indicator with the highest entropy was the CCPT-II omission index (entropy=0.674), being the one with the best discriminating ability for both classes. The Hit RT Block Change indicator had the lowest entropy (entropy=0.199), thus with the lowest power to discriminate groups. The other indexes presented the following entropy contributions: Variability=0.622; Hit RT ISI=0.52; CBCL attention problems=0.481; Hit RT=0.414.

### Best class solution and convergent/divergent validity

A regression analysis was performed to identify if some variables could be powerful predictors of classes in Model 3. Age, gender, type of school, and a clinical diagnosis of ADHD were used in this analysis. Only a clinical diagnosis of ADHD was found to be a strong predictor of the HR-class, (logit=20.87, P<0.001), providing convergent evidence for predicting the classes. The other variables - age (logit=0.08, P=0.89), gender (logit=-0.04, P=0.95), and type of school (logit=0.15, P=0.32) - were not statistically significant, revealing divergent validity.

### FA as a distal outcome of latent class using Lanza's method

After the sample was best rearranged into two new classes according to their attentional functioning, DTI images of these individuals were analyzed in order to explore possible neurobiological differences between the classes.

Only 42 of the 58 subjects had their DTI images considered due to quality criteria. These 42 individuals, when compared respecting the new classes, did not differ significantly regarding age (*t*(40)=0.18, P=0.85), gender (χ^2^(1)=0.06, P=0.81), or IQ (*t*(40)=–1.97, P=0.06), and, therefore, did not interfere with the analysis of the DTI images (see more details in Supplementary Table S2).

The new classes were compared using TFCE statistical analysis in TBSS. Under FWE correction, no results suggested significant differences between the groups. Subsequently, they were compared with respect to the mean FA values of the 20 fiber tracts obtained from the ROI analysis. As presented in Table 3, for each FA mean value, the mean value for each latent class, its standard error (SE), chi-squared overall test comparing the latent classes via Lanza's method, and its respective P values were considered. The Benjamini-Hochberg correction significance level for the 20 P values was 0.00526 and, under this correction, two FA remained statistically significant: the left inferior fronto-occipital fasciculus (IFOF) and left inferior longitudinal fasciculus (ILF). As can be seen in Table 3, the HR-class presented increased FA mean values for these two fiber tracts.

**Table 3:** Comparison of latent classes in respect to fractional anisotropy (FA) mean values.

FA	HR-class Mean (SE)	ND-class Mean (SE)	Overall χ^2^ test	P value
Anterior thalamic radiation L	0.37 (0.07)	0.44 (0.02)	0.90	0.343
Anterior thalamic radiation R	0.48 (0.01)	0.39 (0.03)	0.01	0.011
Corticospinal tract L	0.43 (0.08)	0.47 (0.06)	0.64	0.637
Corticospinal tract R	0.55 (0.02)	0.52 (0.02)	0.33	0.332
Cingulum (cingulate gyrus) L	0.48 (0.02)	0.47 (0.02)	0.52	0.516
Cingulum (cingulate gyrus) R	0.40 (0.05)	0.43 (0.02)	0.27	0.601
Cingulum (hippocampus) L	0.41 (0.05)	0.49 (0.03)	0.90	0.904
Cingulum (hippocampus) R	0.40 (0.06)	0.42 (0.03)	0.70	0.793
Forceps major	0.54 (0.03)	0.52 (0.03)	0.19	0.662
Forceps minor	0.57 (0.01)	0.50 (0.03)	4.34	0.037
Inferior fronto-occipital fasciculus L	0.50 (0.01)	0.39 (0.04)	9.77	0.002
Inferior fronto-occipital fasciculus R	0.38 (0.07)	0.44 (0.03)	0.51	0.473
Inferior longitudinal fasciculus L	0.46 (0.02)	0.35 (0.03)	8.95	0.003
Inferior longitudinal fasciculus R	0.47 (0.05)	0.45 (0.02)	0.43	0.514
Superior longitudinal fasciculus L	0.32 (0.07)	0.36 (0.03)	0.11	0.738
Superior longitudinal fasciculus R	0.36 (0.06)	0.40 (0.02)	0.36	0.551
Uncinate fasciculus L	0.33 (0.07)	0.40 (0.03)	0.94	0.333
Uncinate fasciculus R	0.49 (0.05)	0.44 (0.02)	0.43	0.513
Superior longitudinal fasciculus (temporal part) L	0.44 (0.03)	0.48 (0.02)	1.28	0.258
Superior longitudinal fasciculus (temporal part) R	0.27 (0.05)	0.29 (0.04)	0.07	0.795

L: left; R: right; HR-class: ultra-high risk class; ND-class: normal development of attentional functions; SE: standard error.

## Discussion

The main goal of this study was to reclassify two groups of ADHD-I and TD individuals into new classes according to their similar attentional profiles, and then investigate if there were neurobiological differences between the new groups in relation to brain white matter.

Three possible models consisting of cognitive and behavioral attentional measures were built, and Model 3 with a two-class solution was selected as the one that best reorganized the sample, rearranging subjects into two new classes: HR and ND. It is worth pointing out that the sample originally comprised an equal number of individuals from each diagnostic group (i.e., 50.0% ADHD-I; 50.0% TD), but when subjects were reclassified according to cognitive and behavioral variables, only 33.1% of the entire sample were classified as part of the class with an ultra-high risk of attentional problems (HR).

Each class comprised individuals with more similar attentional profiles. Some of the constructs, such as Omissions, Variability, and Hit RT ISI, proved to be more sensitive than others in discriminating the classes. According to a meta-analysis of many versions of the CPT paradigm, omission errors are one of the most consistently impaired measures among individuals with attentional deficits (30). Variability and Hit RT ISI are related to the ability to maintain a consistent response time throughout tasks. Response time variability is also one of the most robust ADHD deficits, and is considered a core feature of the disorder (31). In line with this, individuals with the most impaired attentional features (HR-class) differentiated from other individuals (ND-class) in terms of performance consistency.

Although the classes differed in respect to their attentional profiles, it is noticeable that the ND-class performed better in all measures considered. This effect could pose some questions about the rearranging criteria of the classes. At first sight, it could seem that participants were clustered according to symptom severity. However, considering that about 36.6% of ADHD-I individuals showed a higher probability to be classified as belonging to the ND-class, we assumed that the two-class solution allowed the identification of different attentional profiles among ADHD-I subjects. Moreover, approximately 32% of the ND-class presented 6 or more inattention symptoms according to SNAP-IV, which suggested that although they had some inattentive symptoms, they had a normal attentional profile according to the measures considered here. Thus, ADHD individuals clustered in different classes may have differed in respect to their deficit patterns (in some domains and not in others) and not necessarily in respect to their deficit severity. Indeed, ADHD individuals can present typical performance even in measures that are commonly impaired in the disorder (6).

Many authors have defended the existence of multiple neuropsychological profiles in the disorder (1,4). It seems that each of the many neuropsychological dimensions that have been proposed to be associated to ADHD is impaired among only a subset of individuals with the disorder (1), with some of the measures having substantial distributional overlap between ADHD and TD subjects (6). As argued by Nigg et al. (6), it is possible that samples previously classified as the same ADHD subtype actually consisted of more subgroups of symptomatic children (i.e., with different deficit patterns), as seems to be the case with the ADHD-I sample in the present study.

### Neurobiological data

Our findings revealed that individuals with worse performance in attentional cognitive and behavioral measures showed increased diffusion of water molecules – higher FA values – in two fiber tracts, namely the left IFOF and left ILF. These tracts make up the long association bundles, being considered white matter's main neural pathways, connecting the frontal, temporal, and occipital lobes, i.e., providing the connection between the anterior and posterior brain regions.

According to the attentional model proposed by Petersen and Posner (9), anterior-posterior areas of the brain are responsible for modulating the vigilance network, which is related to alertness and sustained attention. Many studies state that attentional frontoparietal networks play an important role in selective attention and, thus, abnormalities in these networks can be responsible for deficits in this cognitive domain (32).

Although attention relies on a wide network, in their attentional model, Petersen and Posner (9) indicated that the right hemisphere underlies the initiation and maintenance of arousal. Indeed, growing evidence from neuropsychology, neuroanatomy, and neurochemistry supports the idea that the right hemisphere plays an important role in attention functioning (33). In line with this finding, Riccio et al. (34) stated that the role of the right hemisphere in performance in CPT tasks is evident across multiple studies, reiterating the important role of this hemisphere in the maintenance of attention. As the majority of the attentional measures adopted in our analysis were extracted from a visual paradigm test – CCPT-II –, which required both attentional control and implicit visual processing, we assume that adequate performance in this task would depend on the unimpaired functioning of the right anterior-posterior cortical areas.

However, as already mentioned above, in our study we found that the HR-class presented white matter abnormalities in tracts from the left hemisphere. Initially, this finding might bring our results into question, but it should be noted that although increased FA values are usually associated with higher white matter integrity or more myelination of a fiber tract, when this data is found in pathological samples, it can be the result of a reduction in fiber branching in regions where more fiber branching should actually exist, or be due to compensatory mechanisms (11), as a reduced integrity in locally dominant fiber could result in an increased net directional preference of water diffusion within white matter in perpendicular tracts (13). In many studies, these increased FA values are explained by compensatory mechanism, even though they were not necessarily accompanied by a reduction in fractional anisotropy values of other fibers (13). Thus, in our results, increased FA values in the left hemisphere tracts may be due to the inappropriate development of the IFOF and ILF tracts in the right hemisphere.

In their occipital course, these two tracts run together and are part of the sagittal stratum (SS). Peterson et al. (13) pointed out that increased FA values in the sagittal stratum were correlated with inattentive symptomatology, which is consistent with our results, since the HR-class showed higher FA values in two of the three tracts that compose the SS.

The IFOF connects the dorsal, medial occipital, and parietal regions with the caudodorsal prefrontal cortex (35). The IFOF conveys reciprocal connections between these areas, being one of the networks responsible for vigilance maintenance and visual processing (36). Moreover, the IFOF is concerned with higher top-down spatial aspects of attention (37), playing an important role in visual-spatial processing and in attention control (35,38). Considering the importance of this tract for appropriate attentional performance, we can infer that the HR-class' worse performance in attentional measures – drawn from a visual paradigm attentional task – might be associated with less effective branching of this tract in the right hemisphere, which may subsequently have resulted in increased FA values in the left IFOF for these subjects due to compensatory mechanisms.

The ILF is a ventral associative bundle with long and short fibers connecting the occipital and temporal lobes (39). The left ILF is also part of the visual sensory attentional network, influencing visual search tasks (40). Previous evidence has identified correlations between DTI measures (medium diffusivity) of the ILF and reaction time variability in the CPT, highlighting the role of this tract as part of the sensory visual attentional network. In line with this, in our results, subjects with a worse performance in reaction time variability in the CCPT-II (HR-class participants) presented abnormalities in the left ILF.

We can, therefore, hypothesize that if the right hemisphere fiber tracts responsible for attention control and visual-spatial processing are not functioning properly in the HR-class, the left hemisphere tracts – IFOF and ILF – may have partially assumed their processing through compensatory mechanisms. This rearrangement may have led to increased axonal packing of these left hemisphere fibers, and, thus, help to explain the higher FA mean values for IFOF and ILF fibers in the HR-class.

Considering the cognitive and behavioral profiles of individuals as the starting point and relating them to white matter abnormalities seemed to be a more sensitive approach to understand anatomo-functional relationships. As mentioned before, a previous analysis conducted with the same sample of the current study (14) compared the FA values of the same 20 fiber tracts, grouping the subjects according to their original diagnosis group (ADHD-I *vs* TD). Contrasting with the current results, in the previous study no significant differences were found between the groups. Thus, through this classical categorical approach, no white matter differences between the groups were found, even after having selected ADHD individuals with the same subtype, which would allow a greater uniformity within the groups (see reference 14 for more details). However, when a more refined analysis of the possible pathophysiological mechanisms of dysfunction was carried out, arranging participants according to observable cognitive and behavioral measures, neurobiological differences were found.

The differences between the findings of the dimensional (current) analysis and the categorical (previous) analysis gave further support to the idea that brain-behavior relationships may not respect the arbitrary limits imposed by diagnostic classifications, and seemed to vary in accordance with homogenous phenotypes.

Although the present study restricted the sample diversity, comprising only ADHD-I and control individuals, the findings indicated the existence of distinct neuropsychological patterns among the participants, consonant with the conclusions of Fair et al. (1) and van Hulst et al. (4). Moreover, our investigation went beyond the identification of neuropsychological and behavioral profiles, integrating neurobiological data into the analysis. White matter abnormalities found in the IFOF and ILF in the HR-class shed light on neurobiological characteristics that might be associated with inattention. Of course, these results cannot be considered as conclusive, and it would be premature to suggest that these same neurobiological traits could be found in all individuals with a cognitive profile similar to the HR-class. Notwithstanding, our results reiterate the importance of linking cognitive and behavioral variables to neurobiological systems and going beyond a categorical diagnosis, as recommended by RDoC framework. Performance in cognitive tasks can be used as a marker that may contribute to a better identification of subgroups of a given disorder and its underlying biological mechanisms, allowing clinicians to better understand individual phenotypes.

The limitations of this study involve the use of cognitive variables extracted from one attentional task only. As no more indexes could be added to the LCA due to the size of the sample, the replication of the current analysis with a larger sample would allow more cognitive and behavioral indicators to be included. A larger sample would probably permit participants to be regrouped in more classes, and, therefore, allow the identification of a greater diversity of attention profiles among subjects. The small size of our sample also restricts the generalization of our findings.

Our results do not negate the clinical need for diagnostic categories or the existence of the categories themselves but highlight how the heterogeneity of a disorder reflects the need to produce evidence based on cognitive, behavioral, brain, and genetic features. The newly established sub-groups can respond differently to specific interventions and, in the long term, efforts to identify groups of individuals with more homogenous characteristics may be helpful in their treatment and management.

## Supplementary material

Click here to view [pdf].
